# Climate Change as a Health Security Threat in Pakistan: Preparing Health Systems for Extreme Events

**DOI:** 10.5334/aogh.5294

**Published:** 2026-07-30

**Authors:** Tayyab Mansoor Akhtar, Hashaam Akhtar, Babar Tasneem Shaikh, Zahra Hassan

**Affiliations:** 1 Global Health Department, Health Services Academy, Islamabad, Pakistan; 2 School of Public Health, Health Services Academy, Islamabad, Pakistan; 3 National Disaster Management Authority, Islamabad, Pakistan

**Keywords:** climate change, global health security, health systems, Pakistan, Universal health coverage

## Abstract

Pakistan is confronting the climate crisis as an immediate and systemic threat to national health security rather than an environmental concern. Recurrent floods between 2022 and 2025 affected more than 26 million people, damaged health infrastructure, disrupted essential services, and placed the country at the top of the Germanwatch Climate Risk Index. These shocks have intensified pre-existing health system fragilities by driving surges in malaria, waterborne infections, malnutrition, antimicrobial resistance, and forced displacement, while constraining routine service delivery. Climate-related migration to peri-urban informal settlements has created new epidemiological vulnerabilities characterized by overcrowding, poor sanitation, and outbreaks such as extensively drug-resistant typhoid. Simultaneously, crop losses and food system disruption have worsened child undernutrition in districts already exceeding emergency thresholds, with women and children disproportionately affected because of structural barriers to maternal, neonatal, and immunization services. Climate variability is also increasing the risk of zoonotic spillover and undermining progress toward Universal Health Coverage. We argue that climate change functions as a threat multiplier for Pakistan’s health security and must be systematically integrated into health policy and planning. Key priorities include climate-resilient health infrastructure, strengthened integrated surveillance using digital and geospatial tools, prevention-oriented primary care, and operationalization of a One Health framework. Effective intersectoral governance, provincial implementation, and sustained collaboration with international partners, including the World Health Organization, are essential for building a resilient health system capable of maintaining continuity of care during increasingly frequent climate shocks.

Pakistan now stands on the frontline of the climate crisis. Climate change has shifted from being a distant environmental concern to an immediate threat to global health security [1]. From the cold Himalayas in the north to the arid deserts in the south, the country features 42 mountain peaks above 7200 meters above sea level. This extreme vertical distance between the highest peaks and sea level is immense and its diverse climate conditions [2], including heatwaves, waterborne disease burdens, food insecurities, and displacements due to flooding or droughts. The recent devastating floods of 2025. which killed nearly 1000 people in Pakistan and shifted the country from eighth to first position in the Germanwatch Climate Risk Index 2025 for most climate-vulnerable countries [3], are a distressing reminder of the massive and escalating impact of climate change on human, animal, and environmental health. The floods from 2022 to 2025 have affected almost 63 million people in the country [4] and damaged 13% of the medical facilities, with damage exceeding USD 30 billion, including USD 14.9 billion in infrastructure damage and USD 15.2 billion in lost GDP, and leaving behind approximately 15% of the country’s population deprived of basic health facilities [5]. These situations continue to stress the health system throughout the year, not only impacting and exacerbating the country’s existing socioeconomic challenges but also serving as a significant threat multiplier. Pakistan has the highest number of disaster-displaced people after Afghanistan. This paper explores the critical issue of climate change and its profound impact on Pakistan’s health security, underscoring the need for urgent action to enhance health system preparedness and readiness in extreme conditions.

Health security has previously been linked to infectious disease outbreaks, epidemics, endemics, and pandemics, but now climate-induced events are escalating these risks [6]. Climate change is an old term, but its consequences, such as floods, changing rainfall patterns, and heatwaves, have had devastating effects on livelihoods but are now making our lives even more difficult by producing a climate-adapted breed of mosquitoes and other microorganisms, leading to a surge in vector-borne and water-borne diseases around the world [7]. In Pakistan, the 2022 floods were followed by a sharp increase in dengue cases, with major outbreaks reported in Sindh and Khyber Pakhtunkhwa, overwhelming already fragile healthcare facilities. Similarly, waterborne infections such as cholera, diarrhea, and typhoid surged due to contaminated water supplies [8]. The country’s malaria cases increased fivefold in 2022, from 500,000 in 2021 to 2.6 million in 2022, because of catastrophic flooding [4]. Whereas several countries have declared themselves malaria-free, proving that elimination is possible with strong health systems and political will, Pakistan’s progress has been severely set back by recurrent climate shocks. The frequent and unnecessary use of medicines, including antibiotics, combined with a lack of access to clean water and fragile healthcare systems, risks overwhelming healthcare at any time. Climate change exacerbates this crisis further; floodwaters carry antibiotic-resistant microbes from agricultural runoff and livestock waste into drinking water sources, accelerating the spread of antimicrobial resistance (AMR) and complicating treatment of common infections. This interconnected web of climate-sensitive health threats demands an integrated response that addresses both infectious disease outbreaks and the underlying environmental drivers that fuel them.

The rollout of malaria vaccines offers new hope, yet vaccine hesitancy remains a significant challenge to our public health efforts. Pakistan lacks reliable data on vaccine acceptance, making it harder to plan effective immunization drives. As a result, preventable diseases continue to burden an already strained health system. Climate events compound this challenge by disrupting routine vaccination services. The availability of routine vaccination services was reported to be diminished in 2022 in regions impacted by floods. Since Pakistan is one of the top 10 countries in the world with the largest number of children who are either under- or unvaccinated, more than 600,000 youngsters have not received a single dose of vaccination. In Pakistan’s flood-affected districts, there were approximately 650,000 pregnant women, and up to 73,000 of them were thought to have been in labor or delivery shortly before the flooding [9]. This suggests a serious deficiency in the prenatal and postnatal care these women most likely received, as well as a lack of trained birth attendants.

The 2022 floods in Sindh not only directly destroyed health facilities but also triggered a cascade of secondary crises. For instance, the stagnant water in many areas led to a severe surge in malaria cases, Cholera/diarrhea outbreaks, and raised skin-related diseases [9], overwhelming the primary healthcare centers that were already operating at half-capacity due to infrastructure damage. This created a vicious cycle where disease burden further strained a crippled system, exemplifying the “threat multiplier” effect in a tangible, local context. The country’s malaria cases increased fivefold in 2022, from 500,000 in 2021 to 2.6 million in 2022, because of the catastrophic flooding in Pakistan [4]. In contrast, several countries have declared themselves malaria-free, proving that elimination is possible with strong health systems and political will. The rollout of malaria vaccines offers new hope, yet vaccine hesitancy remains a significant challenge to our public health efforts. Pakistan lacks reliable data on vaccine acceptance, making it harder to plan effective immunization drives. As a result, preventable diseases like malaria continue to burden an already strained health system.

Migration from rural flood-affected areas toward urban centers creates a new class of vulnerable urban poor, often settling in squatter settlements *(katchi abadis)* with no access to civic amenities, including health services and sewerage systems, leading to more endemics like polio or epidemics like Cholera, Typhoid or Hepatitis A and E, etc. This internal migration places immense strain on urban health systems, dents immunization tracking systems, and creates pockets of unaddressed disease burden, such as the resurgence of XDR-Typhoid in peri-urban Karachi, linked to overcrowding and poor sanitation [10]. Health security strategies must prioritize these groups by ensuring equitable access to healthcare, social protection, and emergency relief. Climate events disproportionately impact vulnerable, underprivileged, and marginalized groups, especially women and children, who face structural barriers to accessing maternal, neonatal, and immunization services.

Rising temperatures and erratic rainfalls can also disrupt the food system and drives food insecurity worsening the child malnutrition as highlighted in 2022 floods, where 14% of the children in flood-affected districts suffered from severe acute malnutrition, a life-threatening form of malnutrition [11], in a country where 40% of the children below 5 years of age are stunted and almost one in three children are underweight [12]. Furthermore, prolonged heatwaves, particularly in urban centers like Karachi and Lahore, have increased the burden of heatstroke and cardiovascular strain among vulnerable populations. The destruction of millions of acres of cropland exacerbated pre-existing malnutrition [13], particularly among children, especially in flood-affected districts of Sindh and Baluchistan, surpassing emergency thresholds, pushing an already fragile nutritional status into a full-blown humanitarian crisis.

The nation’s efforts to achieve Universal Health Coverage (UHC) are hampered by the climate catastrophe, which also puts a further burden on the already precarious healthcare system. Health Security in Pakistan, therefore, requires adaptation of climate risks into health Policies, strengthening disease surveillance, and building climate-resilient health systems [4]. The zoonotic threat in Pakistan is not merely hypothetical. Climate change has driven habitat relocation and altered human–animal interfaces, multiplying the risk of zoonotic spillover. For instance, Crimean-Congo Hemorrhagic Fever (CCHF), transmitted by ticks that thrive in warmer temperatures, has seen increased case reports in Balochistan and Punjab following periods of drought followed by rainfall. Similarly, leptospirosis outbreaks have been documented in flood-affected urban areas of Sindh, where contaminated floodwater carrying animal urine comes into contact with human populations. Date palm-growing regions in Sindh and Punjab have been impacted by endemic plant viruses. The danger of zoonotic spillover to people and cattle has multiplied due to habitat relocation caused by climate change. A proactive, integrated surveillance system must, therefore, monitor not only human febrile illnesses and livestock health but also environmental parameters such as temperature, rainfall patterns, humidity, and vegetation indices, which serve as early warning signals for disease emergence [14]. For example, tracking abnormal rainfall and temperature spikes could predict tick population surges and trigger pre-emptive vector control measures before human cases emerge. This integrated approach—linking climate data with epidemiological and veterinary surveillance—is a critical, yet under-implemented, national security imperative. As discussed earlier, climate change exacerbates another silent pandemic, that is, AMR. The rampant, unregulated use of antibiotics in agriculture, poultry, and livestock sectors is compounded by floodwaters that spread antibiotic-resistant microbes from farms and waste into water supplies. High prevalence of multi-drug-resistant *Escherichia coli* in flood-affected urban water sources is no doubt an environmental disaster and a public health threat [15]. A One Health approach would operationalize surveillance across three domains: human clinical laboratories tracking resistance patterns, veterinary and agricultural monitoring of antibiotic use in livestock, and environmental sampling of water sources for resistance genes. This triangulated data would enable early detection of emerging resistance hotspots and inform targeted interventions, such as restricting antibiotic use in specific livestock operations or treating contaminated water sources before outbreaks occur. Addressing these interconnected challenges requires a One Health framework that links human, animal, and environmental health to build stronger disease prevention and response systems [16].

The best course of action may be to evaluate and adapt public health program design to reflect climate threats for both communicable and noncommunicable illnesses [17]. Pakistan’s escalating healthcare burden can be mitigated by adopting preventative care and healthy living practices, as seen in many Western health systems. Treatment expenses can be reduced by up to $10 for every dollar spent on prevention. Embedding prevention-focused strategies into primary care and climate policies is essential for a climate-resilient health system. Climate events repeatedly disrupt services, highlighting the need for climate-sensitive planning. Disaster preparedness should include early warning systems, regular drills, and community awareness, along with accessible healthcare, psychosocial support, and affordable insurance for recovery [18]. Researchers and public health experts must advocate for integrating health security into climate adaptation policies. Pakistan should prioritize climate-resilient healthcare infrastructure that ensures uninterrupted services and withstands extreme weather.

Aligning with World Health Organization (WHO) guidance, systems must be sustainable and reduce carbon footprint [19]. The use of digital tools, including artificial intelligence (AI), data analytics, and geospatial systems, can significantly strengthen surveillance and early warning capabilities [20]. However, this presents a notable paradox: AI and high-performance computing are themselves major consumers of energy. To reconcile this, Pakistan must invest in renewable-energy-powered data centers and energy-efficient algorithms, ensuring that technological advancement does not inadvertently exacerbate the carbon footprint of the health sector.

Equally critical is the clinical health sector, which must be equipped to recognize and manage climate-sensitive health conditions. Medical and nursing curricula in Pakistan currently lack comprehensive training on climate-related health issues. We recommend integrating climate health modules into undergraduate and postgraduate medical education, covering topics such as heatstroke management, vector-borne disease diagnosis, malnutrition screening, and mental health first response following climate disasters. Continuing professional development programs should also be established to upskill practicing clinicians. Training community health workers to serve as first responders during climate emergencies, identifying early warning signs, triaging patients, and linking affected populations to health facilities would further strengthen the frontline response. This human capacity building, combined with infrastructure resilience and sustainable technology, forms the foundation of a health system capable of withstanding the escalating climate crisis.

Climate change disproportionately impacts women, particularly in settings shaped by restrictive gender norms. Limited mobility can prevent timely evacuation during floods and restrict access to essential services, including emergency obstetric care. During droughts, women and girls often travel longer distances to fetch water, increasing their exposure to heat-related illness and gender-based violence. To address these vulnerabilities, Pakistan must implement gender-responsive climate and health policies with concrete measures. For example, mobile health units should be deployed to flood-affected areas specifically to provide antenatal and postnatal care, ensuring that pregnant women with restricted mobility still receive essential services. Female community health workers (Lady Health Workers) should be equipped with climate-specific training and emergency communication tools to serve as first responders in their communities. Early warning systems should be designed with gender-segregated messaging, for instance, ensuring that evacuation alerts reach women through trusted channels such as lady health workers or community-based organizations, rather than solely through male-dominated village councils. Social protection programs, such as cash transfers for climate-affected families, should be directly disbursed to women to enhance their financial autonomy and decision-making power in emergencies. Global and multilateral actors such as WHO and the World Bank’s Strategic Climate Fund play a critical role in addressing climate-related health risks in Pakistan, alongside strengthened health diplomacy for knowledge sharing and technology transfer [4]. For Pakistan, this translates into tangible support: the World Bank’s Climate Risk and Adaptation Country Profile provides data that can inform province-level health adaptation planning; WHO’s operational framework for climate-resilient health systems offers a blueprint for strengthening infrastructure and surveillance; and the Green Climate Fund has resources that could be leveraged to finance solar-powered health facilities in off-grid flood-prone districts. However, accessing these resources requires strengthened health diplomacy, knowledge sharing, and technology transfer to ensure that international commitments translate into on-the-ground improvements [4]. While Pakistan’s National Climate Change Policy 2021 exists, health remains insufficiently integrated, with challenges including fragmented funding, weak inter-ministerial coordination, and varying provincial capacity [21]. For instance, the policy lacks specific health sector adaptation targets, such as the percentage of health facilities that must be climate-proofed by 2030 or the proportion of the health budget allocated to climate resilience. A coordinated, health-centered policy response with province-specific implementation plans and clear accountability mechanisms is urgently needed to prepare for climate shocks, reduce inequities, and safeguard progress toward UHC and global health security.

## References

[r1] Pakistan GIZ. Pakistan: On the front line of climate change. Published 2024. Accessed April 7, 2026. https://www.giz.de/en/downloads_els/Pakistan_%20On%20The%20Front%20Line%20of%20Climate%20Change.pdf.

[r2] Zafar U, Anjum MN, Hussain S, et al. Analyzing the spatiotemporal changes in climatic extremes in cold and mountainous environment: Insights from the Himalayan Mountains of Pakistan. Atmosphere. 2024;15(10):1221. doi:10.3390/atmos15101221.

[r3] Germanwatch. German Watch 2025 Climate Risk Index: Who suffers most from extreme weather events?. Published 2025. Accessed July 10, 2026. https://www.germanwatch.org/sites/default/files/202502/Climate%20Risk%20Index%202025.pdf.

[r4] National Disaster Management Authority. Post Monsoon Review 2025. Published 2025. Accessed April 7, 2026. https://www.ndma.gov.pk/storage/publications/December2025/NUgyCMG8uMfYsFhVMXka.pdf.

[r5] Akthar TM, Ried MJA. The urgency of climate-resilient health systems in Pakistan: Lessons from the 2022 floods. Int J Public Health. 2024;69:1607981. doi:10.3389/ijph.2024.1607981. 39545056 PMC11563755

[r6] Semenza JC, Rocklöv J, Ebi KL. Climate change and cascading risks from infectious disease. Infect Dis Ther. 2022;11(4):1371–1390. doi:10.1007/s40121-022-00647-3. 35585385 PMC9334478

[r7] Zhao Q, Yu P, Mahendran R, et al. Global climate change and human health: Pathways and possible solutions. Eco Environ Health. 2022;1(2):53–62. doi:10.1016/j.eehl.2022.04.004. 38075529 PMC10702927

[r8] Saeed HA, Abbas SW. Study of dengue outbreak during 2022 floods among patients presenting to a tertiary level hospital in Nowshera, Pakistan. Pak Armed Forces Med J. 2025;75(1):32–35. doi:10.51253/pafmj.v75i1.9764.

[r9] Abdullah MA, Shaikh BT, Sikander A, Sarwar B. Public health and health system’s responsiveness during the 2022 floods in Pakistan: What needs to be done? Disaster Med Public Health Prep. 2024;17:e567. doi:10.1017/dmp.2023.224. 38163991

[r10] Butt MH, Saleem A, Javed SO, et al. Rising XDR-typhoid fever cases in Pakistan: Are we heading back to the pre-antibiotic era? Front Public Health. 2022;9:794868. doi:10.3389/fpubh.2021.794868. 35111719 PMC8801676

[r11] Center for Disaster Philanthropy. 2022 Pakistan Floods. Published 2023. Accessed April 7, 2026. https://disasterphilanthropy.org/disasters/2022-pakistan-floods/.

[r12] Akhtar TM, Jalal M, Khaliq MA. Malnutrition in children: A national security threat for Pakistan. Indus J Biosci Res. 2025;3(10):1–2. doi:10.70749/ijbr.v3i10.2615.

[r13] Akhtar TM, Khaliq AM, Habib MF. Feeding Pakistan in a changing climate. Int J Health Sci. 2025;9(S1):514–516.

[r14] Yasmeen N, Jabbar A, Shah T, et al. One Health paradigm to confront zoonotic health threats: A Pakistan prospective. Front Microbiol. 2022;12:719334. doi:10.3389/fmicb.2021.719334. 35211097 PMC8861076

[r15] Khan M, Khan S, Basharat S. Exacerbating crises: The devastating impact of climate change and antibiotic resistance on human and animal health in Pakistan. J Coll Physicians Surg Pak. 2025;35(9):1203–1206. doi:10.29271/jcpsp.2025.09.1203. 40948172

[r16] Abdullah MA, Shaikh BT. Pathways to One Health: Enhancing inter-sectoral collaboration in Pakistan. Ecohealth. 2025;22(1):138–146. doi:10.1007/s10393-024-01696-5. 39755986

[r17] Fox M, Zuidema C, Bauman B, Burke T, Sheehan M. Integrating public health into climate change policy and planning: State of practice update. Int J Environ Res Public Health. 2019;16(18):3232. doi:10.3390/ijerph16183232. 31487789 PMC6765852

[r18] Khan M, Muhammad F, Bibi M, Tayyab M. Climate risk reduction, disaster preparedness and flood resilience in Pakistan. Khyber J Public Policy. 2024;3(4):1–26. https://nipapeshawar.gov.pk/KJPPM/PDF/CIP/41PSS/PSS10.pdf.

[r19] World Health Organization. Building climate-resilient health systems. Published 2026. Accessed April 7, 2026. https://www.who.int/teams/environment-climate-change-and-health/climate-change-and-health/country-support/building-climate-resilient-health-systems.

[r20] Ali SH, Shahid R. Artificial intelligence for climate resilience: The case of Pakistan. Islamabad Policy Res Inst. 2025;XXV(2):151–180. doi:10.31945/iprij.250208.

[r21] Government of Pakistan. Updated Nationally Determined Contributions 2021. Published 2021. Accessed April 7, 2026. https://unfccc.int/sites/default/files/NDC/2022-06/Pakistan%20Updated%20NDC%202021.pdf.

